# Supplementary material for: ACE2-containing defensosomes serve as decoys to inhibit SARS-CoV-2 infection
Source: PLoS Biol. 2022 Sep 13;20(9):e3001754. doi: 10.1371/journal.pbio.3001754 (PMC9469972; doi:10.1371/journal.pbio.3001754)
Supplement: S1 Raw images — (PDF) [file pbio.3001754.s005.pdf]

S1 Raw Images

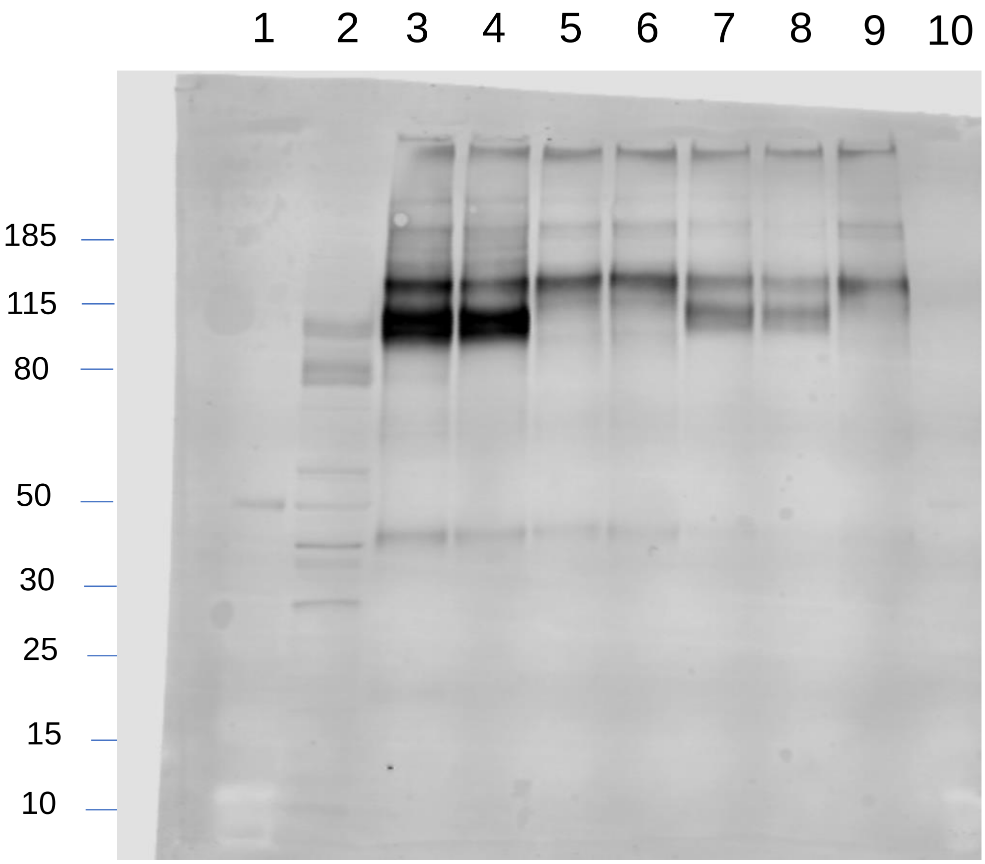

Lanes/Sample

1. Pageruler plus protein ladder
2. HAEC cell lysate
3. ACE2+ A549 exosome lysate (baf induced)
4. ACE2+ A549 exosome lysate (IFNα induced)
5. dACE2+ A549 exosome lysate (baf induced)
6. dACE2+ A549 exosome lysate (IFNα induced)
7. Calu3 exosome lysate (baf induced)
8. Calu3 exosome lysate (IFNα induced)
9. Untransduced A549 exosome lysate (Baf induced)
10. Pageruler plus protein ladder

- Primary Ab: goat anti-ACE2 R&D AF933
- Blot imaged on a Licor Odyssey CLX Imaging System
- Used to generate Supplemental Figure 6

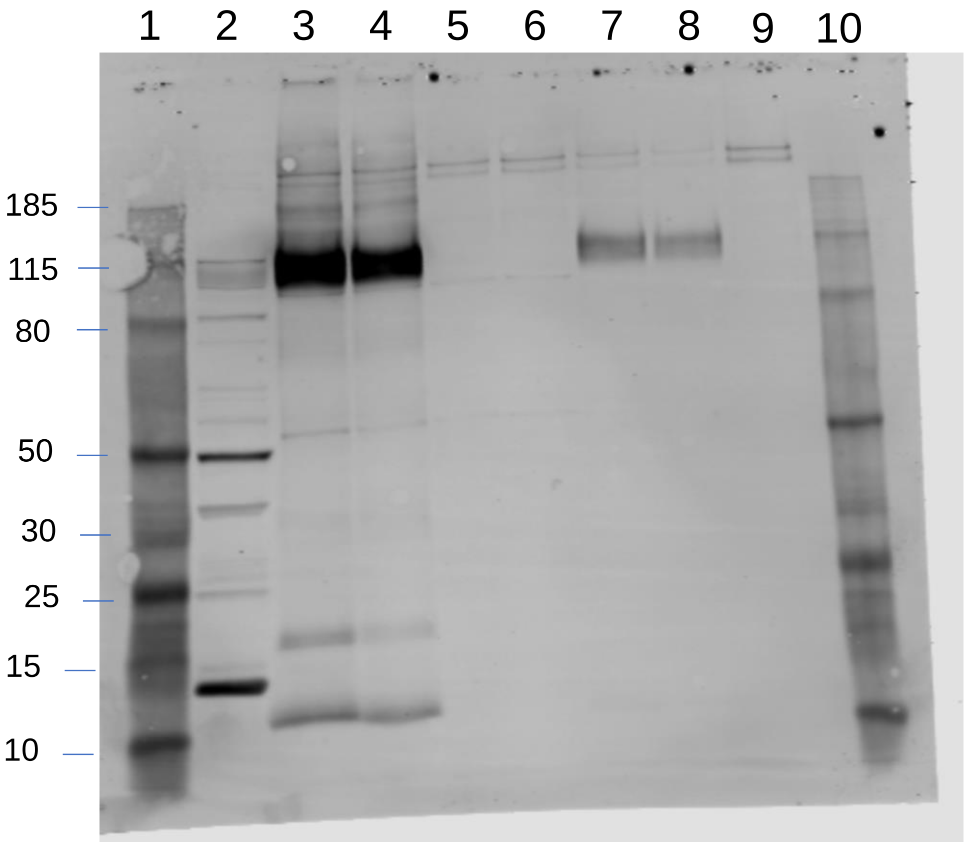

Lanes/Sample

1. Pageruler plus protein ladder
2. HAEC cell lysate
3. ACE2+ A549 exosome lysate (baf induced)
4. ACE2+ A549 exosome lysate (IFNα induced)
5. dACE2+ A549 exosome lysate (baf induced)
6. dACE2+ A549 exosome lysate (IFNα induced)
7. Calue3 exosome lysate (baf induced)
8. Calu3 exosome lysate (IFNα induced)
9. Untransduced A549 exosome lysate (Baf induced)
10. Pageruler plus protein ladder

- Primary Ab: Abcam Rb anti-ACE2 (ab15348), monoclonal mouse anti-CD9 (R&D, cat# MAB25292)
- Blot imaged on a Licor Odyssey CLX Imaging system
- Used to generate Supplemental Figure 6

S1 Raw Images

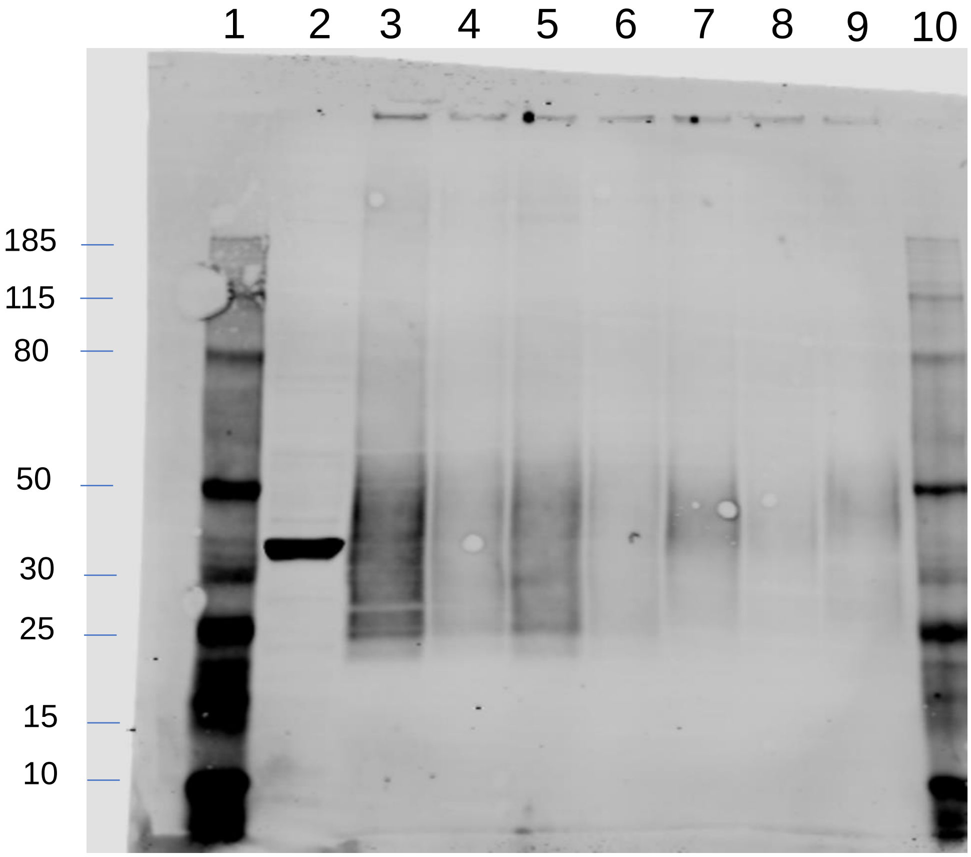

Lanes/Sample

1. Pageruler plus protein ladder
2. HAEC cell lysate
3. ACE2+ A549 exosome lysate (baf induced)
4. ACE2+ A549 exosome lysate (IFNα induced)
5. dACE2+ A549 exosome lysate (baf induced)
6. dACE2+ A549 exosome lysate (IFNα induced)
7. Calu3 exosome lysate (baf induced)
8. Calu3 exosome lysate (IFNα induced)
9. Untransduced A549 exosome lysate (Baf induced)
10. Pageruler plus protein ladder

- Primary Ab: Abcam mouse anti-CD63 (ab217345)
- Blot imaged on a Licor Odyssey CLX Imaging System
- Used to generate Supplemental Figure 6
