# Supplementary figures and images for: ACE2-containing defensosomes serve as decoys to inhibit SARS-CoV-2 infection
Source: PLoS Biol. 2022 Sep 13;20(9):e3001754. doi: 10.1371/journal.pbio.3001754 (PMC9469972; doi:10.1371/journal.pbio.3001754)

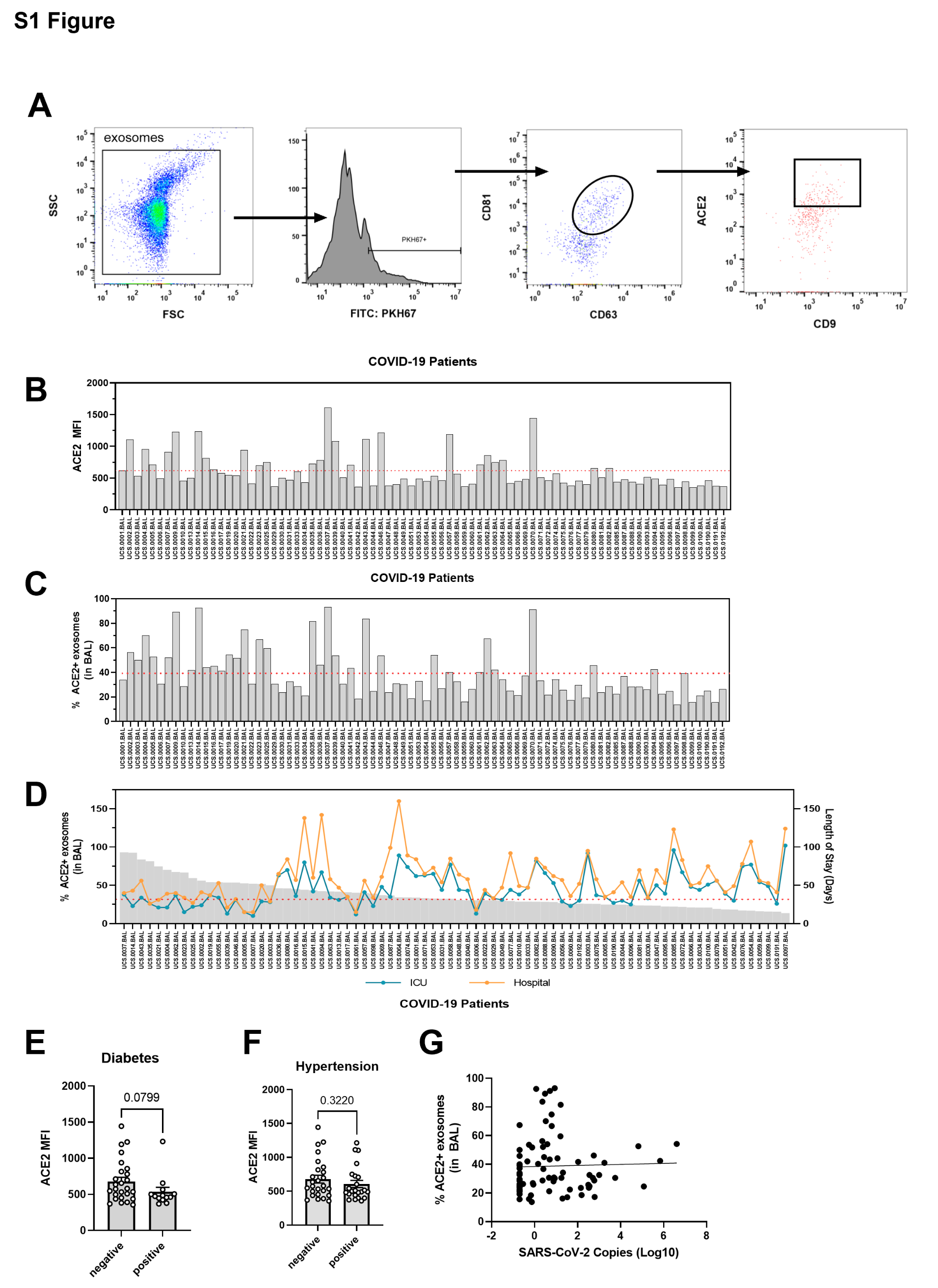

Supplement: S1 Fig — (A) Gating strategy and representative flow cytometry plots from patient BALF. Exosomes were stained for antibodies against CD63, CD9, CD81, and ACE2. Exosomes were labeled with a lipid-intercalating dye, PKH67. FSC: forward scatter. SSC: side scatter. (B) Surface ACE2 on exosomes isolated from acellular BALF measured by MFI using flow cytometry. (C) Percentage of ACE2+ exosomes out of total exosomes in A. (D) Correlation between percentage of ACE2+ exosomes and length of stay in the ICU (orange line) and hospital (teal line) for all patients (including deaths) (N = 80). ACE2 levels on exosomes stratified by positive or negative patient status for (E) diabetes and (F) hypertension (N = 80). (G) Correlation between viral RNA copies and percent ACE2+ exosomes (N = 80). Red dotted lines indicate the average ACE2 MFI on ACE2+ exosomes in B or the average proportion of ACE2+ exosomes in C calculated from all COVID patients. Error bars show mean ± SEM. Underlying data can be found in S1 Data. E, F Unpaired Mann–Whitney t test. G Simple linear regression.* P ≤ 0.05; ** P ≤ 0.01; *** P ≤ 0.001; **** P ≤ 0.0001. BALF, bronchoalveolar lavage fluid; COVID 19, Coronavirus Disease 2019; ICU, intensive care unit; MFI, mean fluorescence intensity; ns, not significant. (TIFF) [file pbio.3001754.s006.tiff]

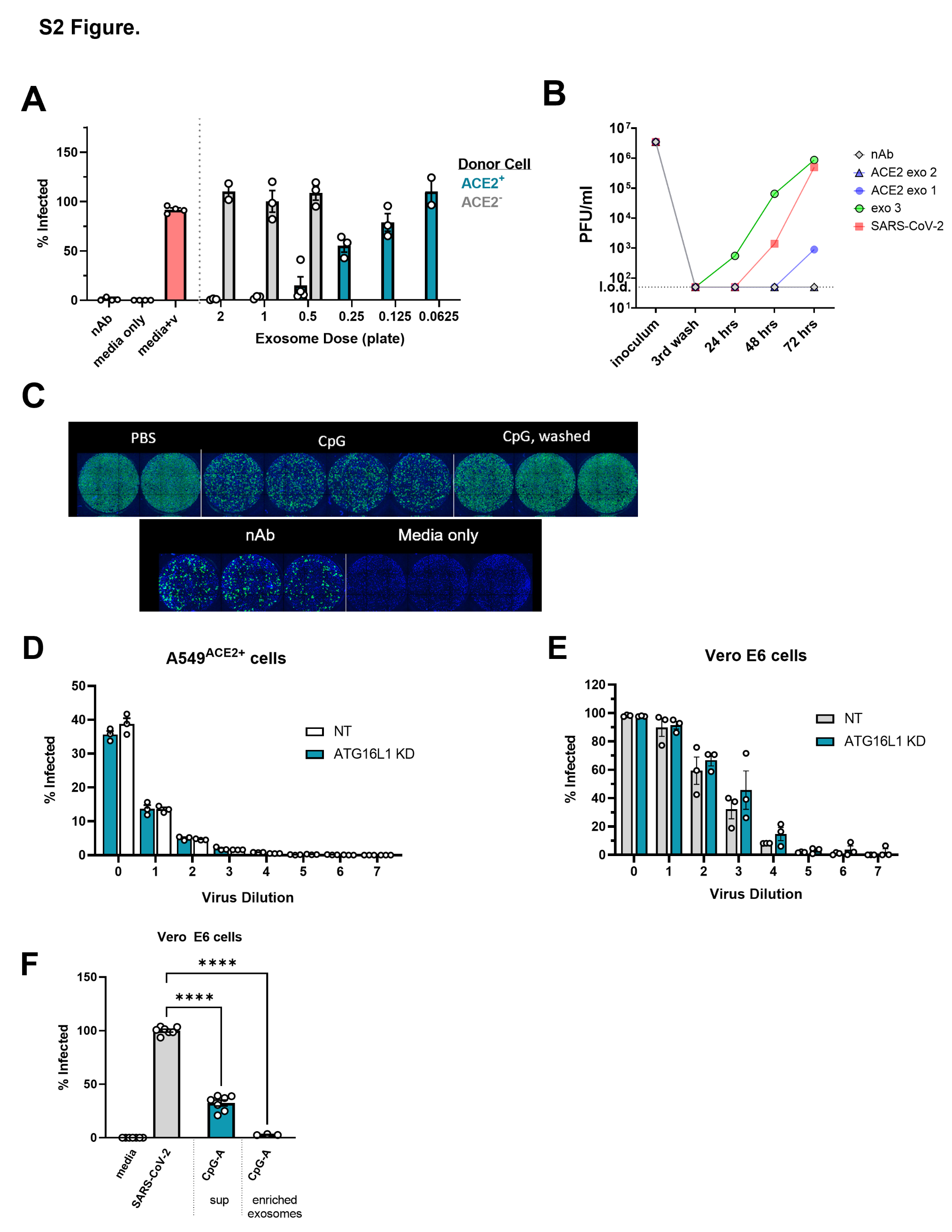

Supplement: S2 Fig — (A) Infection level in Fig 3D against number of donor A549ACE2+ or A549 cells (in 15 cm plates). (B) Number of infectious particles measured by plaque assay from apical washes every 24 h for 72 h of HAECs infected with SARS-CoV-2 alone (red), SARS-CoV-2 and ACE2+ exosomes (blue), SARS-CoV-2 and ACE2− exosomes (green), and SARS-CoV-2 with neutralizing Ab (gray); # corresponds to number of input plates (15 cm). (C) Representative immunofluorescence images of Vero E6 cells infected with SARS-CoV-2 where the cells were stimulated/pretreated with PBS, CpG, or nAb from Fig 3F. Scale bar: 1.25 mm. (D) Infection level 24 hpi with SARS-CoV-2 of ATG16L1 KD or non-targeting shRNA control (NT) A549ACE2+ cells. (E) Infection level 24 hpi with SARS-CoV-2 in ATG16L1 KD or NT control Vero E6 cells. Virus dilution in (D) and (E) corresponds to the step in a 2-fold dilution series of SARS-CoV-2 (MOI: 0.01). (F) Infection level of Vero E6 cells following infection with media alone, SARS-CoV-2 alone, or SARS-CoV-2 mixed with supernatant from Vero E6 cells stimulated with CpG-A or enriched exosomes from CpG-A stimulated Vero E6 cells. Error bars show mean ± SEM, with measurements taken from distinct samples. Underlying data can be found in S1 Data. F One-way ANOVA with Dunnett’s post-test compared to SARS-CoV-2. **** P ≤ 0.0001. HAEC, human airway epithelial culture; hpi, hours post infection; KD, knockdown; ns, not significant; SARS‑CoV‑2, Severe Acute Respiratory Syndrome Coronavirus 2. (TIFF) [file pbio.3001754.s007.tiff]

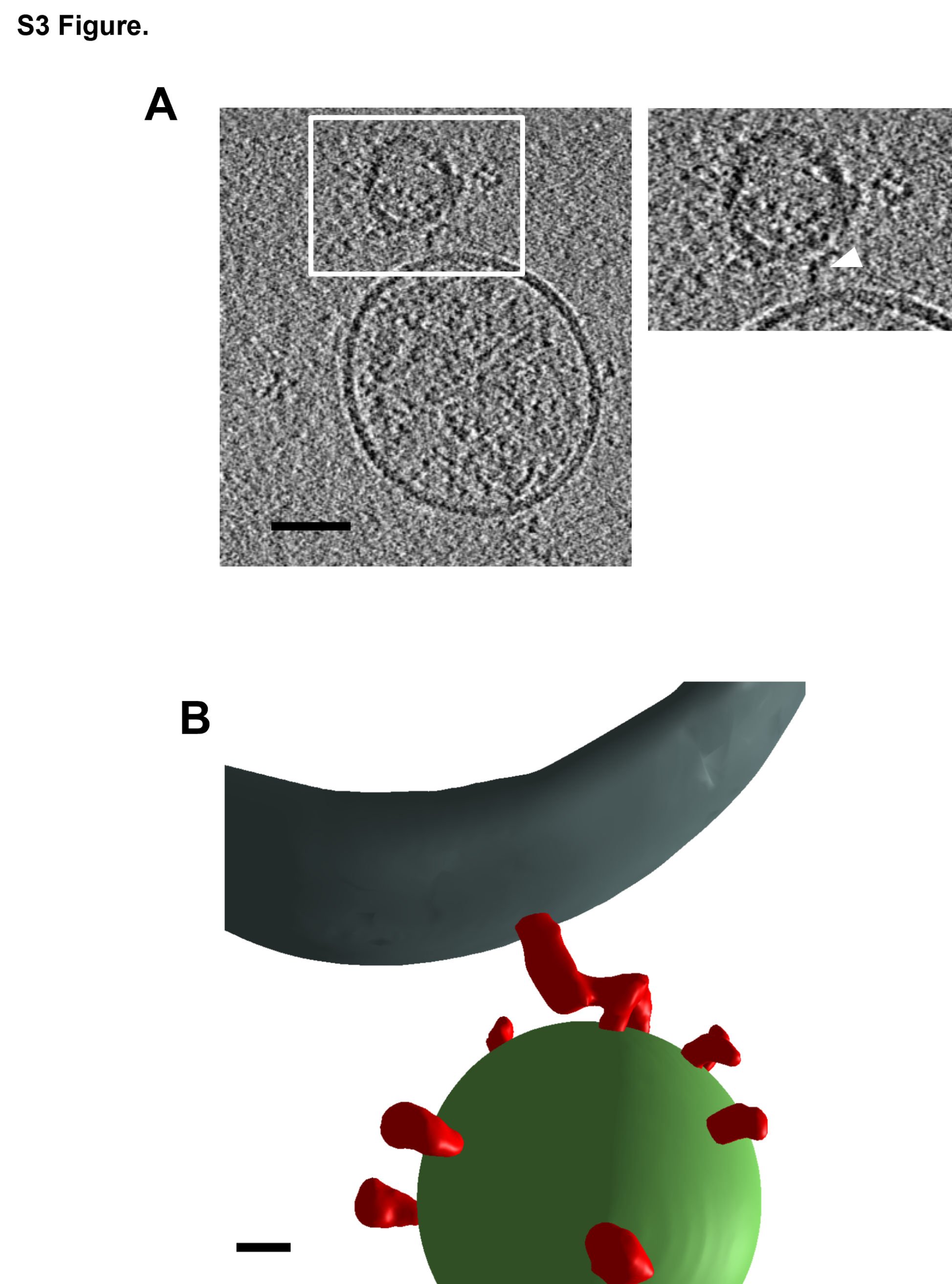

Supplement: S3 Fig — (A) Representative tomographic slice of a SARS-CoV-2 virion and an exosome. Exosomes were isolated from ACE2+ A549 cells. Inset white arrow: spike, scale bar: 50 nm. (B) Three-dimensional model of 1 individual exosome (gray) and SARS-CoV-2 virion with a membrane (green) and spike proteins (red) generated from segmentation. Scale bar: 10 nm. SARS‑CoV‑2, Severe Acute Respiratory Syndrome Coronavirus 2. (TIFF) [file pbio.3001754.s008.tiff]

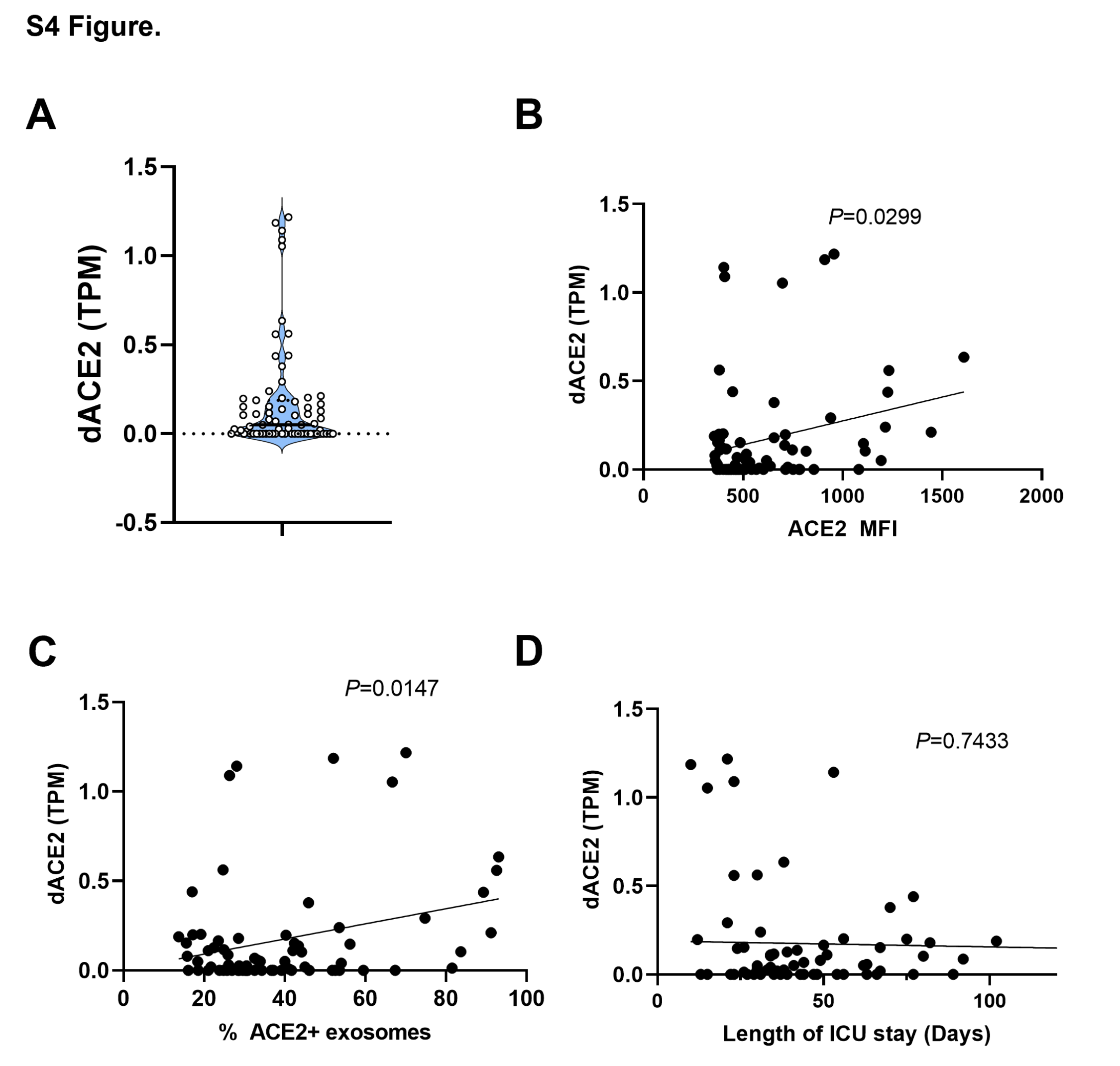

Supplement: S4 Fig — (A). Normalized expression of dACE2 measured by RNA-seq (TPM) from the cellular fraction of BAL fluid isolated from hospitalized COVID patients (N = 70). (B). dACE2 expression correlated against ACE2 MFI (C) % ACE2+ exosomes and (D) length of stay in the ICU (N = 70). Underlying data can be found in S1 Data. ** P ≤ 0.01; **** P ≤ 0.0001. ICU, intensive care unit; MFI, mean fluorescence intensity; ns, not significant; SARS‑CoV‑2, Severe Acute Respiratory Syndrome Coronavirus 2; TPM, transcript per million. (TIFF) [file pbio.3001754.s009.tiff]

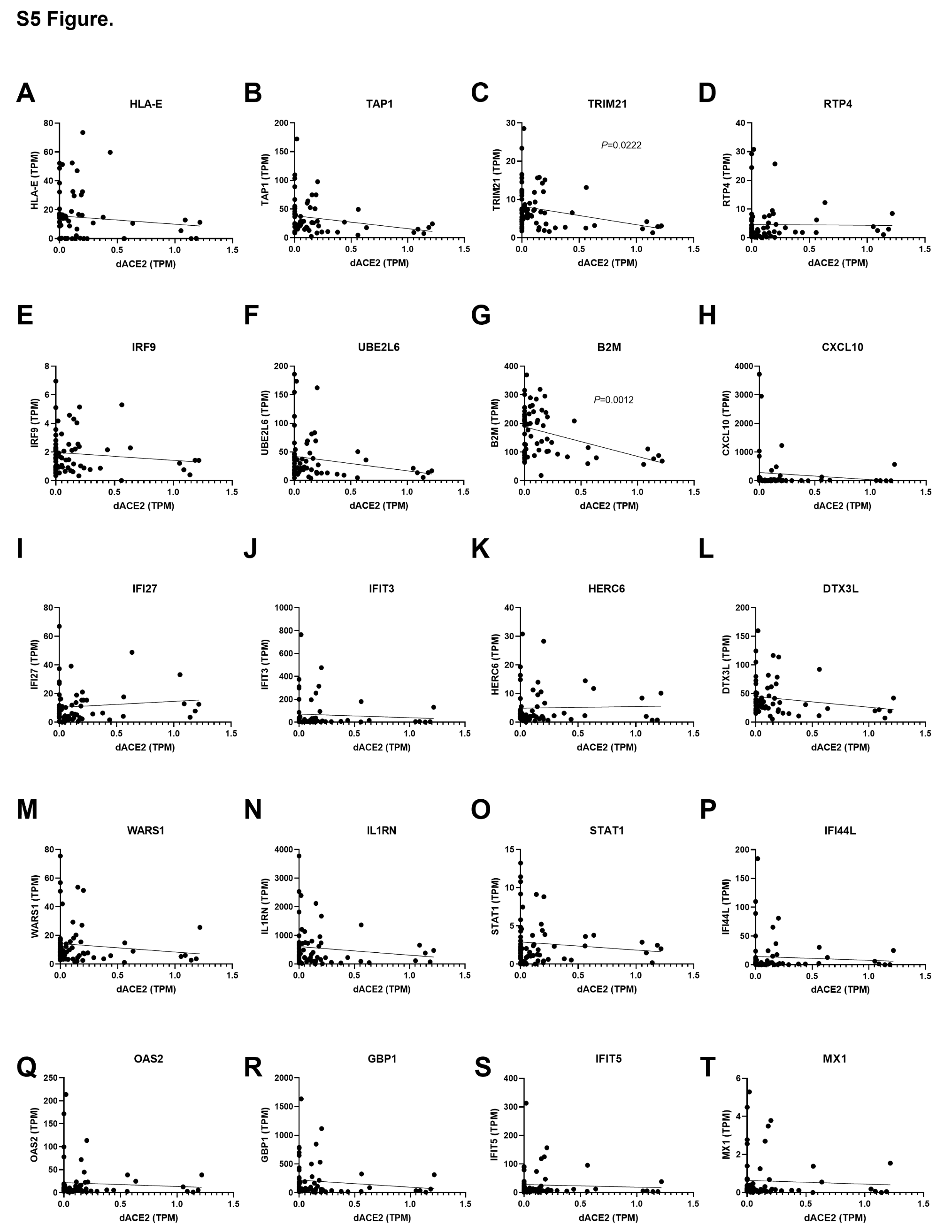

Supplement: S5 Fig — (A–T) Correlation analysis of dACE2 and HLA-E (A), TAP1 (B), TRIM21 (C), RTP4 (D), IRF9 (E), UBE2L6 (F), B2M (G), CXCL10 (H), IFI27 (I), IFIT3 (J), HERC6 (K), DTX3L (L), WARS1 (M), IL1RN (N), STAT1 (O), IFI44L (P), OAS2 (Q), GBP1 (R), (S), and MX1 (T) expression in the cellular fraction of BAL fluid from COVID patients. Samples were excluded if transcriptome mapping was lower than 60% (N = 67). Simple linear regression was performed for A–T. Each data point represents a patient. * P ≤ 0.05; ** P ≤ 0.01; *** P ≤ 0.001; **** P ≤ 0.0001. ns, not significant; TPM, transcript per million. (TIFF) [file pbio.3001754.s010.tiff]

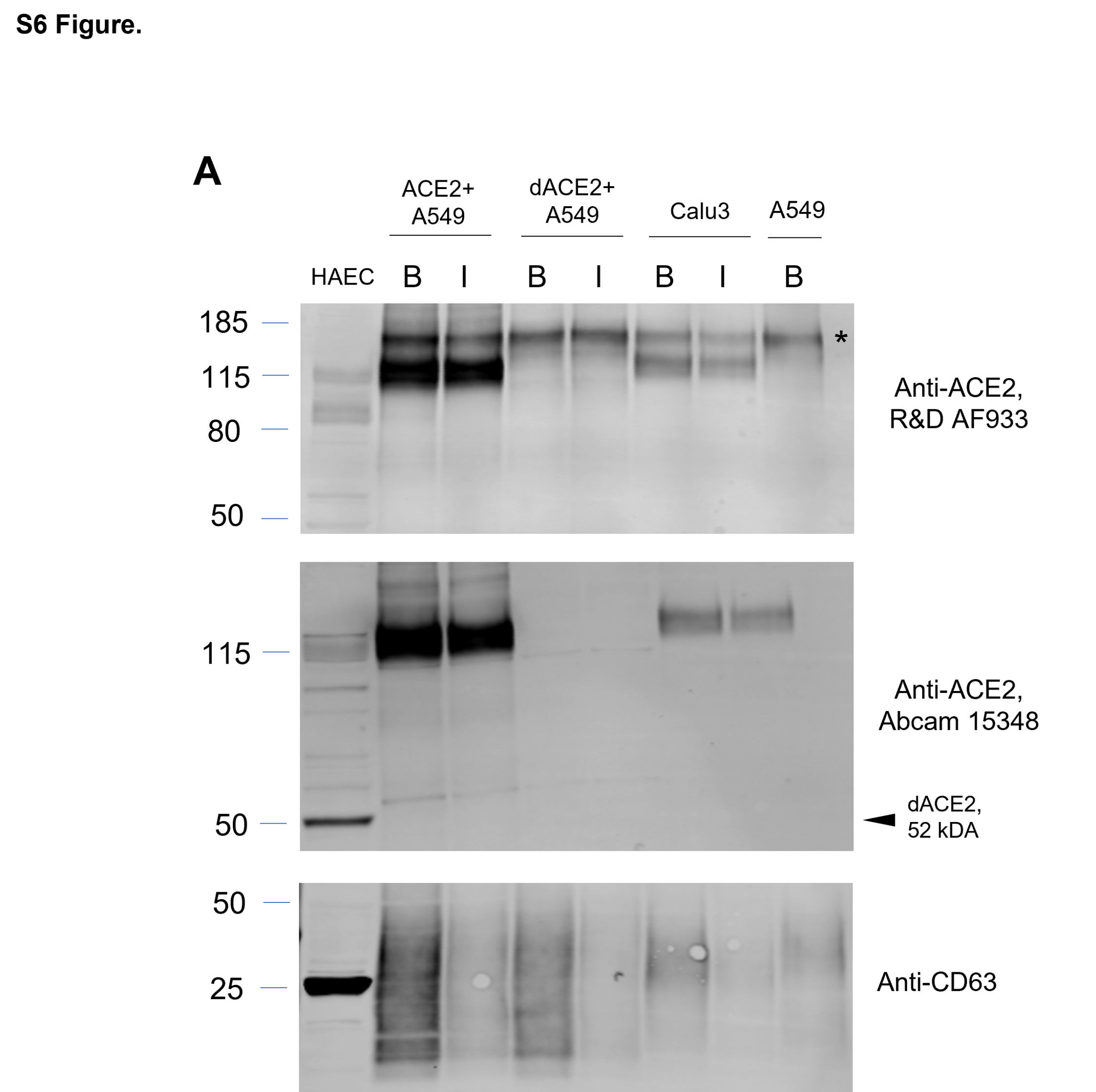

Supplement: S6 Fig — (A) Representative western blots of exosomes from A549 cells expressing full-length ACE2 (ACE2+ A549), dACE2 expressing A549 cells (dACE2+ A549), Calu3 cells, and untransduced A549 cells after stimulation with bafilomycin A1 (B) or IFN-α (I) using 2 different anti-ACE2 antibodies, R&D AF933 (above) and Abcam 15348 (below). R&D AF933 was used for flow cytometry experiments and recognizes full-length ACE2 but not dACE2. Abcam 15348 was raised against the C-terminal region of ACE2 and recognizes both isoforms. Cell lysate from unstimulated HAECs was used as a control for detection of dACE2 protein. dACE2 was not detected in exosome fractions, even when supernatants were from cells ectopically expressing this isoform (dACE2+ A549). Asterisk denotes nonspecific band. HAEC, human airway epithelial culture. (TIFF) [file pbio.3001754.s011.tiff]

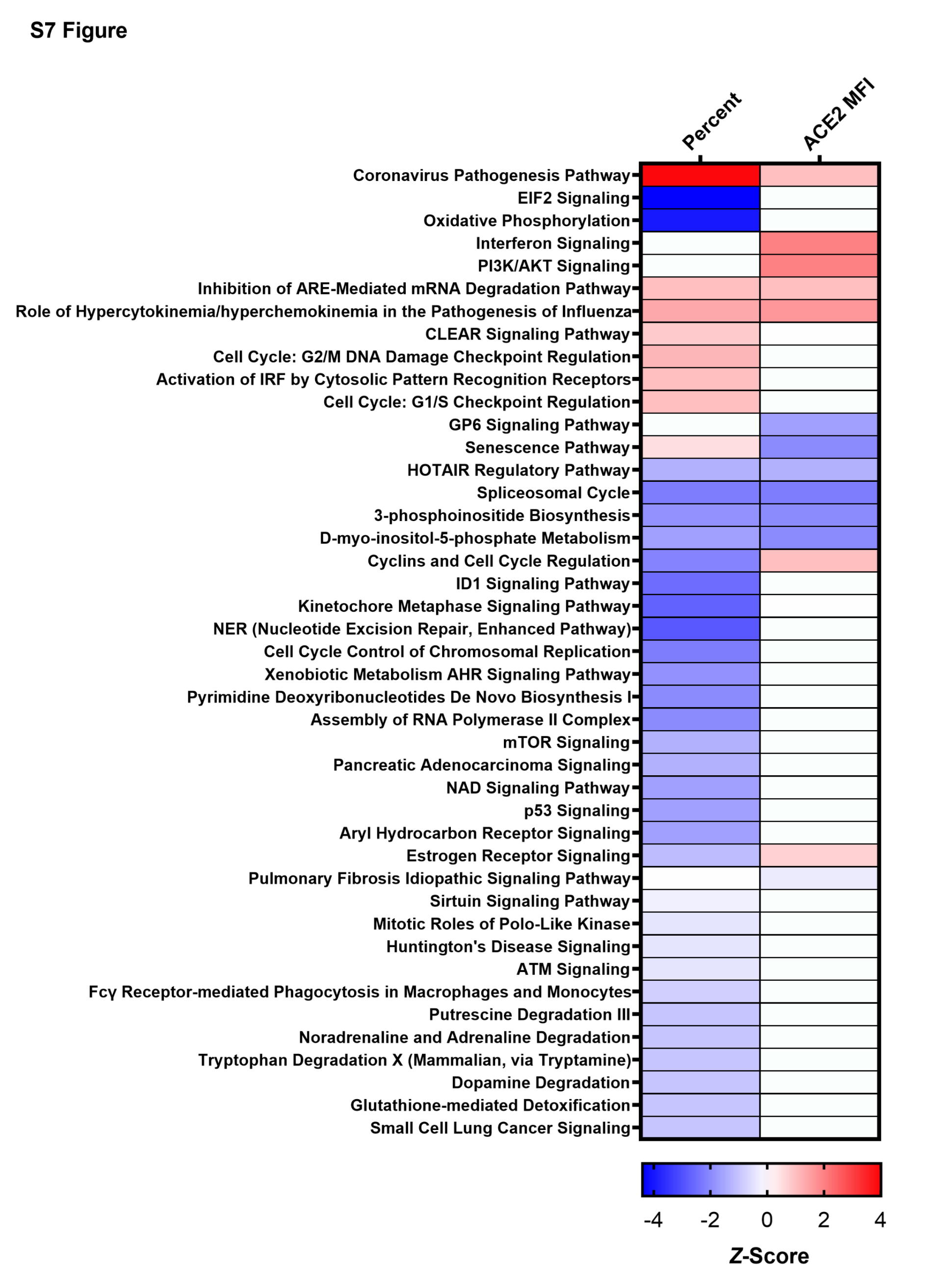

Supplement: S7 Fig — “High” and “low” groups were designated based on whether the measured values of %ACE2+ exosomes and ACE2 MFI for an individual patient were above or below the mean computed value across all patients for each variable. Percent: % ACE2+ exosomes. Red and blue indicate up-regulated and down-regulated pathways in the “high” group for each variable, respectively. IPA, Ingenuity Pathway Analysis; MFI, mean fluorescence intensity. (TIFF) [file pbio.3001754.s012.tiff]

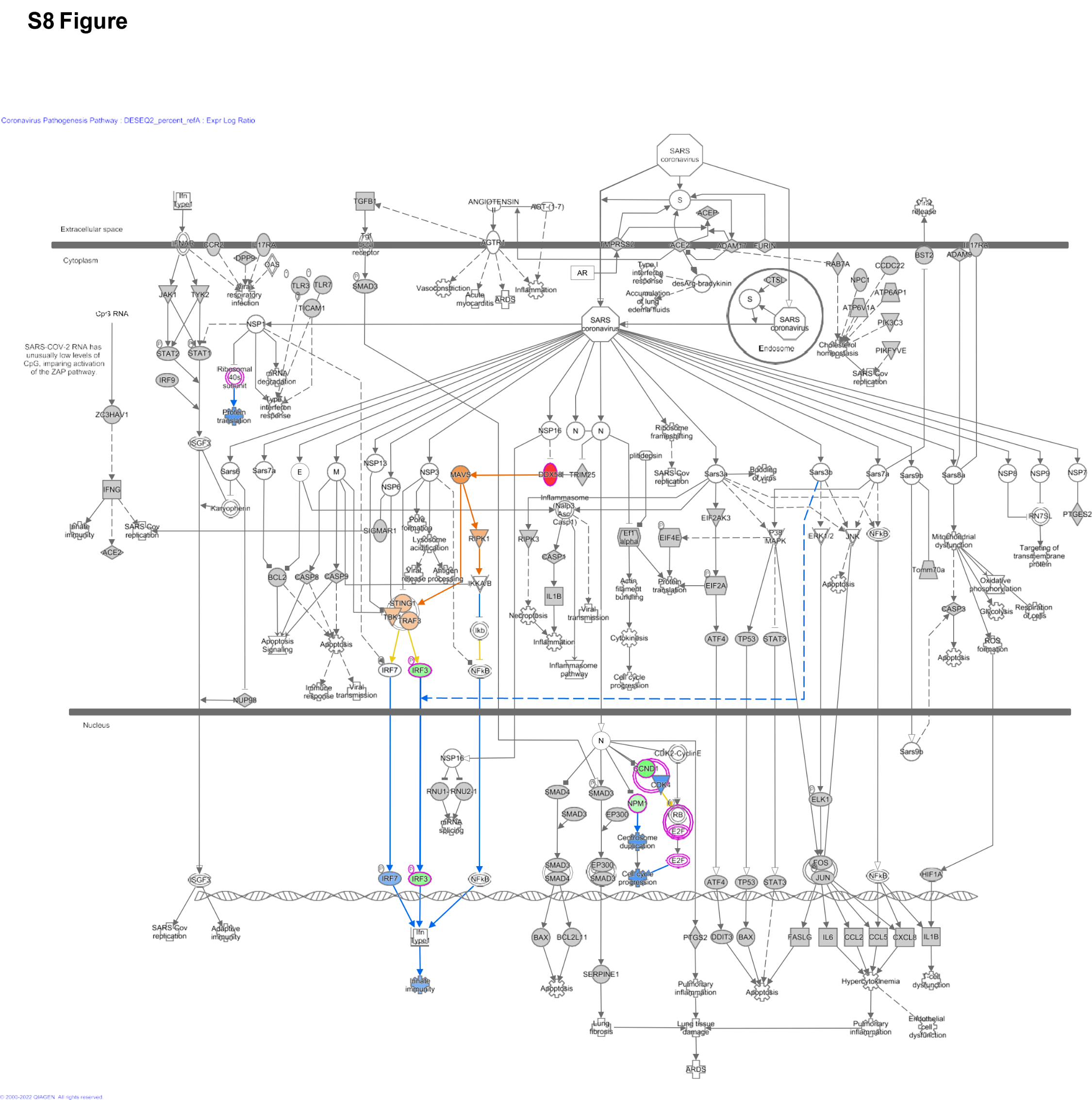

Supplement: S8 Fig — Generated using IPA. IPA, Ingenuity Pathway Analysis. (TIFF) [file pbio.3001754.s013.tiff]

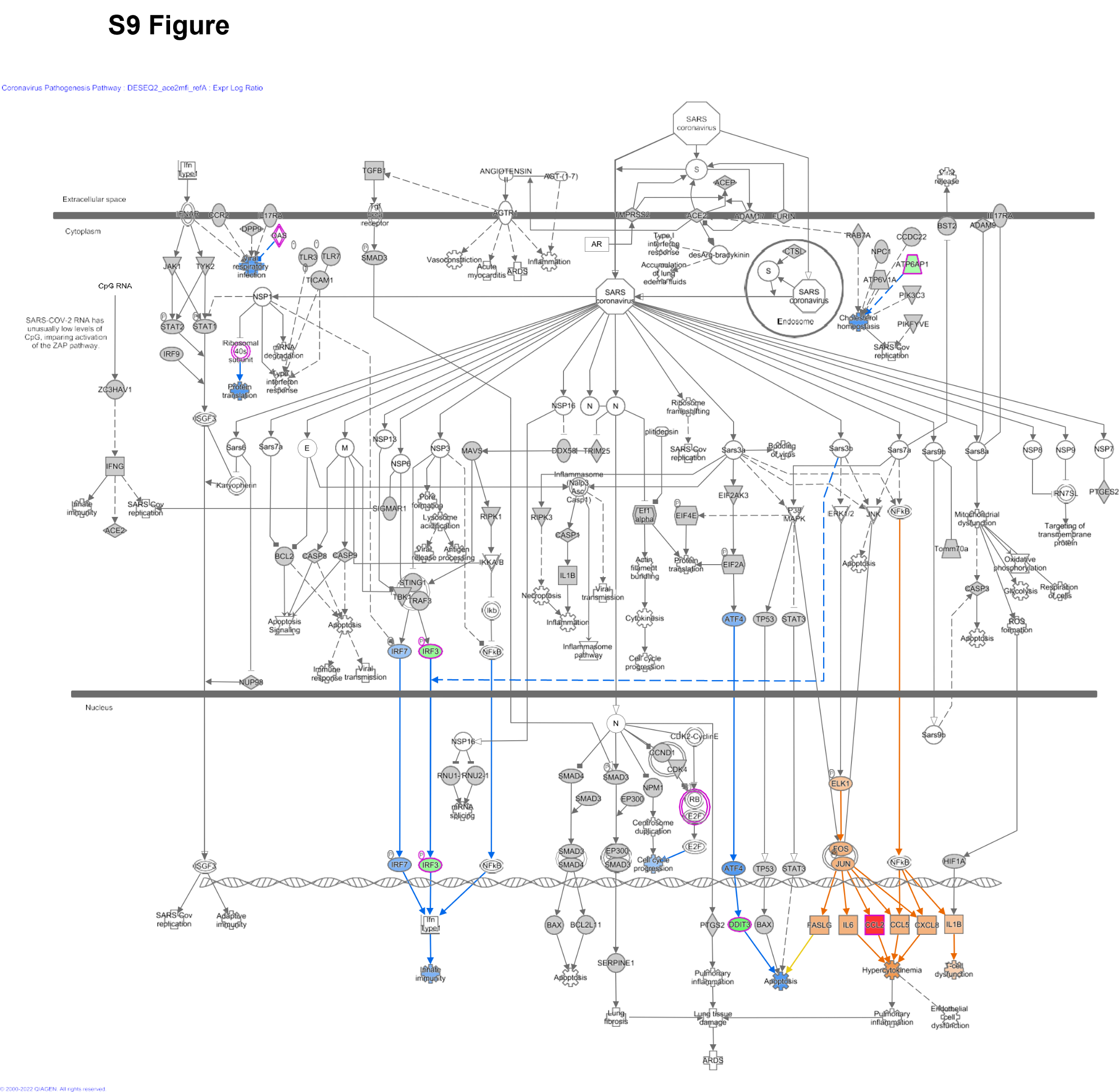

Supplement: S9 Fig — Generated using IPA. IPA, Ingenuity Pathway Analysis; MFI, mean fluorescence intensity. (TIFF) [file pbio.3001754.s014.tiff]
