# Supplementary material for: ACE2-containing defensosomes serve as decoys to inhibit SARS-CoV-2 infection
Source: PLoS Biol. 2022 Sep 13;20(9):e3001754. doi: 10.1371/journal.pbio.3001754 (PMC9469972; doi:10.1371/journal.pbio.3001754)
Supplement: S1 Table — Summary of hospitalized COVID-19 patients from which the ACE2+ exosomes were measured from the BALF. Data expressed as n (%) or median [IQR]. BMI, body mass index. BMI is the weight in kilograms divided by the square of the height in meters. CHF: congestive heart failure; CAD: coronary artery disease; CKD: chronic kidney disease; CVA: cerebrovascular accident. HLD: hyperlipidemia; HTN: Hypertension; ECMO: extracorporeal membrane oxygenation. Respiratory culture: defined as having any respiratory culture performed. Positive bacteria: a culture resulting in any bacterial growth. Blood culture: defined as having any blood culture performed. ICU: intensive care unit; Ventilator days: total number of days on mechanical ventilation. (PDF) [file pbio.3001754.s015.pdf]

S1 Table. Demographics and clinical characteristics of the COVID-19 patient cohort. Summary of hospitalized COVID-19 patients from which the ACE2+ exosomes were measured from the BALF. Data expressed as n(%) or Median[IQR]. BMI, body mass index. BMI is the weight in kilograms divided by the square of the height in meters. CHF: congestive heart failure; CAD: coronary artery disease; CKD: chronic kidney disease; CVA: cerebrovascular accident. HLD: hyperlipidemia; HTN: Hypertension; ECMO: extracorporeal membrane oxygenation. Respiratory culture: defined as having any respiratory culture performed. Positive bacteria: a culture resulting in any bacterial growth. Blood culture: defined as having any blood culture performed. ICU: intensive care unit; Ventilator days: total number of days on mechanical ventilation.

**Variables**	**Cohort**
**N**	80
**Age**	64 [51-71]
**Age Category**	
<51	19 (23.8%)
51-60	11 (13.8%)
61-70	27 (33.8%)
>70	23 (28.7%)
**Sex (Male)**	62 (77.5%)
**Race**	
White	37 (46.2%)
Asian	3 (3.8%)
Black or African American	8 (10%)
Other	32 (40%)
**Ethnicity**	
Hispanic or Latino	21 (26.2%)
**BMI**	26.5 [24-30]
BMI >=30	24 (30%)
**Comorbidities**	
Asthma	1 (1.2%)
CAD	12 (15%)
CHF	6 (7.5%)
CKD	11 (13.8%)
CVA	15 (18.8%)
Diabetes	31 (38.8%)
HLD	26 (32.5%)
HTN	41 (51.2%)
**Smoking Status**	
Never	53 (66.2%)
Ex.Smoker	12 (15%)

Current	6 (7.5%)
**Respiratory Culture**	
Any respiratory pathogenic bacteria	66 (82.5%)
Staphylococcus aureus	15 (18.8%)
C. albicans	17 (21.3%)
**Blood Culture**	
Any positive bacterial culture	46 (57.5%)
**Treatment and Outcomes**	
Intubated	80 (100%)
Dialysis	20 (25%)
Dialysis days	20 [9-32]
ECMO	13 (16.2%)
ECMO days	14 [8-71]
Ventilator days	33 [24-49]
ICU length of stay	39 [30-62]
Hospital length of stay	53 [39-73]
**Mortality**	19 (23.8%)
