# Supplementary material for: ACE2-containing defensosomes serve as decoys to inhibit SARS-CoV-2 infection
Source: PLoS Biol. 2022 Sep 13;20(9):e3001754. doi: 10.1371/journal.pbio.3001754 (PMC9469972; doi:10.1371/journal.pbio.3001754)
Supplement: S2 Table — p, P-value, CI, confidence interval. (PDF) [file pbio.3001754.s016.pdf]

**S2 Table.** Regression using negative binomial model and length of stay in the ICU as the outcome. *p*, P-value, *CI*, confidence interval

| Length of stay in ICU (Days) | | | |
|---|---|---|---|
| ***Predictors*** | *Incidence Rate Ratios* | *CI* | *p* |
| Age (years) | 1.0072 | 0.9907 – 1.0240 | 0.395 |
| % ACE2 positive (BAL exosomes) | 0.9865 | 0.9816 – 0.9915 | **<0.001** |
| Sex [M] | 2.5665 | 0.8587 – 7.6710 | 0.092 |
| Hypertension | 1.9773 | 0.2710 – 14.4269 | 0.501 |
| BAL *C. albicans* [Positive] | 1.5818 | 1.1825 – 2.1159 | **0.002** |
| Blood culture final result [Positive] | 1.1724 | 0.9698 – 1.4173 | 0.1 |
| **(Intercept)** | 46.6348 | 17.3749 – 125.1689 | <0.001 |
| **Observations** | 78 | | |
| **R2 Nagelkerke** | 0.581 | | |
