# Supplementary material for: ACE2-containing defensosomes serve as decoys to inhibit SARS-CoV-2 infection
Source: PLoS Biol. 2022 Sep 13;20(9):e3001754. doi: 10.1371/journal.pbio.3001754 (PMC9469972; doi:10.1371/journal.pbio.3001754)
Supplement: S3 Table — p, P-value, CI, confidence interval. (PDF) [file pbio.3001754.s017.pdf]

S3 Table. Linear regression on covariates including ACE2 MFI using length of stay in the ICU as the outcome. *p*, P-value, *CI*, confidence interval

	Length of Stay in ICU (Days)		
Predictors	*Estimates*	*CI*	*p*
Age (years)	0.4685	-0.3995 – 1.3365	0.285
ACE2 MFI	-0.0291	-0.0481 – -0.0100	**0.003**
sex [M]	53.6830	-3.6455 – 111.0116	0.066
Hypertension	22.1203	-80.3790 – 124.6196	0.668
BAL *C. albicans* [Positive]	21.1636	5.5735 – 36.7537	**0.009**
Blood culture final result [Positive]	4.5791	-5.4529 – 14.6112	0.366
**(Intercept)**	36.1704	-14.3409 – 86.6817	0.158
**Observations**	78		
**R^2 / R^2 adjusted**	0.273 / 0.165		
