# Supplementary material for: ACE2-containing defensosomes serve as decoys to inhibit SARS-CoV-2 infection
Source: PLoS Biol. 2022 Sep 13;20(9):e3001754. doi: 10.1371/journal.pbio.3001754 (PMC9469972; doi:10.1371/journal.pbio.3001754)
Supplement: S4 Table — p, P-value, CI, confidence interval. (PDF) [file pbio.3001754.s018.pdf]

S4 Table. Linear regression on covariates including ACE2 MFI using ventilation days as an outcome. *p*, P-value, *CI*, confidence interval

**Ventilation Days**			
Predictors	*Estimates*	*CI*	*p*
Age (Years)	0.55	-0.42 – 1.51	0.265
ACE2 MFI	-0.01	-0.03 – 0.00	0.132
Sex [M]	70.09	6.37 – 133.82	**0.032**
Hypertension	-41.15	-154.43 – 72.13	0.471
BAL *C. albicans* [Positive]	25.70	9.15 – 42.25	**0.003**
Blood culture final result [Positive]	-2.35	-13.48 – 8.79	0.676
**(Intercept)**	16.34	-39.90 – 72.58	0.564
**Observations**	80		
**R^2 / R^2 adjusted**	0.290 / 0.187		
