# Supplementary material for: ACE2-containing defensosomes serve as decoys to inhibit SARS-CoV-2 infection
Source: PLoS Biol. 2022 Sep 13;20(9):e3001754. doi: 10.1371/journal.pbio.3001754 (PMC9469972; doi:10.1371/journal.pbio.3001754)
Supplement: S5 Table — p, P-value, CI, confidence interval. (PDF) [file pbio.3001754.s019.pdf]

S5 Table. Regression using negative binomial model and ventilation days as the outcome. *p*, P-value, *CI*, confidence interval

Ventilation Days			
Predictors	*Incidence Rate Ratios*	*CI*	*p*
Age (Years)	1.0153	0.9947 – 1.0363	0.146
% ACE2 positive (BAL exosomes)	0.9923	0.9869 – 0.9979	**0.007**
Sex [M]	5.3818	1.4056 – 20.6056	**0.014**
Hypertension	0.4474	0.0416 – 4.8157	0.507
BAL *C. albicans* [Positive]	1.6952	1.2044 – 2.3859	**0.002**
Blood culture final result [Positive]	1.0380	0.8232 – 1.3089	0.752
**(Intercept)**	20.4347	6.0498 – 69.0235	**<0.001**
**Observations**	80		
**R^2 Nagelkerke**	0.483		
